# The Limited Diagnostic Utility of Metabolic and Inflammatory Indices in Adolescent Polycystic Ovary Syndrome: A Case–Control Study

**DOI:** 10.3390/biomedicines14071598

**Published:** 2026-07-16

**Authors:** Sefer Ustebay, Rulin Deniz, Alihan Tigli, Guzide Ece Akinci, Muhammet Bora Uzuner, Nazli Sener, Yasemin Ercan Degirmenci, Dondu Ulker Ustebay, Oğuzhan Karakoç, Yasin Selcuk Yardibi, Deniz Almak, Yakup Baykus

**Affiliations:** 1 Department of Pediatrics, Faculty of Medicine, Bandırma OnYedi Eylül University, 10200 Bandırma, Turkey; sustebay@bandirma.edu.tr (S.U.); yyardibi@bandirma.edu.tr (Y.S.Y.); 2 Department of Obstetrics and Gynecology, Faculty of Medicine, Bandırma OnYedi Eylül University, 10200 Bandırma, Turkey; rdeniz@bandirma.edu.tr (R.D.); atigli@bandirma.edu.tr (A.T.); nsener@bandirma.edu.tr (N.S.); ydegirmenci@bandirma.edu.tr (Y.E.D.); 3 Gynecology and Obstetrics Clinic, Şehit Prof. Dr. İlhan Varank Sancaktepe Training and Research Hospital, 34785 Istanbul, Turkey; geceakinci@gmail.com; 4 Department of Anatomy, Faculty of Medicine, Bandırma OnYedi Eylül University, 10200 Bandırma, Turkey; muzuner@bandirma.edu.tr; 5 Department of Pediatric Neurology, Faculty of Medicine, Bandırma OnYedi Eylül University, 10200 Bandırma, Turkey; dustebay@bandirma.edu.tr; 6 Gynecology and Obstetrics Clinic, Bandırma Training and Research Hospital, 10200 Bandırma, Turkey; oguzhankarakoc1986@hotmail.com; 7 Bandırma No. 1 Family Health Centre, Balıkesir Provincial Health Directorate, 10200 Bandırma, Turkey; dr.denizalmak@gmail.com

**Keywords:** adolescence, polycystic ovary syndrome, insulin resistance, TyG index, inflammation, biomarkers

## Abstract

**Background:** The aim of this study was to evaluate the clinical and diagnostic utility of metabolic and systemic inflammatory marker indices in adolescent patients with polycystic ovary syndrome (PCOS). **Methods**: This retrospective case–control study included 63 adolescent girls diagnosed with PCOS, based on the strict presence of both menstrual irregularity and hyperandrogenism, and 63 healthy controls matched for age and body mass index (BMI). Fasting blood glucose (FBG), fasting insulin (FI), and lipid profiles were measured. Metabolic indices, including the Triglyceride-Glucose (TyG) index, Homeostasis Model Assessment of Insulin Resistance (HOMA-IR), and Metabolic Score for Insulin Resistance (METS-IR)**,** as well as systemic inflammatory indices (Systemic Immune-Inflammation Index (SII), Systemic Inflammation Response Index (SIRI), and the Aggregate Index of Systemic Inflammation (AISI))**,** were calculated. The diagnostic performance of these markers was evaluated using Receiver Operating Characteristic (ROC) curves and binary logistic regression analyses. **Results**: No statistically significant difference was observed between the PCOS and control groups in terms of age and BMI (*p* > 0.05). FBG levels (*p* = 0.001) and the TyG index (*p* = 0.028) were found to be significantly higher in the PCOS group. However, no significant differences were observed between the groups in terms of other metabolic indices (HOMA-IR, METS-IR) and systemic inflammatory markers (SII, SIRI, AISI). In the ROC analysis, only the TyG index demonstrated statistically significant but weak discriminatory power for PCOS (AUC = 0.614; 95% CI: 0.515–0.712; *p* = 0.028). Furthermore, binary logistic regression analysis revealed that the TyG index was not an independent predictor of PCOS after adjustment. **Conclusions**: While early metabolic signals such as elevated FBG and TyG index were detected, the evaluated systemic inflammatory indices did not demonstrate significant differences. However, the weak discriminatory capacity of the TyG index and the failure of other composite indices restrict their clinical utility. Therefore, these biomarkers cannot be recommended as reliable standalone diagnostic tools in adolescent PCOS.

## 1. Introduction

Although polycystic ovary syndrome (PCOS) is one of the most common endocrine disorders in adolescents, its diagnosis still presents significant clinical challenges. This may be primarily explained by the considerable overlap between the key diagnostic features of PCOS, including irregular menstrual bleeding, hirsutism, and polycystic ovarian morphology, and the normal physiological processes of puberty [1,2]. However, PCOS is not merely a reproductive health issue; it is a systemic disorder characterized by significant cardiometabolic risk factors such as insulin resistance, dyslipidemia, and impaired glucose tolerance [3,4]. Early identification of these risks during adolescence is critically important for reducing long-term morbidity through lifestyle interventions [5,6].

Recently, the pathophysiology of PCOS has been described as a multifactorial process in which chronic low-grade inflammation interacts with metabolic disorders [7,8,9]. This complex relationship between insulin resistance, dyslipidemia, and inflammatory activity creates a self-perpetuating cycle; therefore, early risk assessment strategies based on a single laboratory parameter, such as glucose or insulin alone, may be insufficient [10,11]. In this context, there has been growing interest in composite biomarkers that more comprehensively reflect metabolic and inflammatory processes. Metabolic indices derived from lipid parameters, glucose, and insulin levels have been regarded as promising tools for predicting metabolic dysfunction and insulin resistance [12,13]. Likewise, the evaluation of inflammatory markers in conjunction with traditional metabolic parameters may enable a better characterization of cardiometabolic vulnerability. Overall, these multi-parameter approaches are regarded as more powerful tools for risk stratification than isolated biochemical measurements [14].

Various composite indices have been developed to evaluate the inflammatory and metabolic dysfunction that plays central roles in the pathophysiology of PCOS. Among inflammation-based hematological indices, the Systemic Immune-Inflammation Index (SII) is based on a combined assessment of neutrophil, lymphocyte, and platelet counts [15,16], whilst the Systemic Inflammation Response Index (SIRI) includes neutrophil, monocyte, and lymphocyte components. The Aggregate Index of Systemic Inflammation (AISI), by contrast, includes all four parameters and stands out as a more comprehensive indicator of systemic inflammatory burden [17]. These indices are thought to reflect the biological complexity of inflammation more effectively compared to individual markers [18]. Among metabolic markers, the Triglyceride-Glucose (TyG) Index—calculated from triglyceride (TG) and fasting blood glucose (FBG) levels—has gained attention as a practical indicator of insulin resistance [19,20]. Although the Homeostasis Model Assessment of Insulin Resistance (HOMA-IR), based on fasting insulin (FI) and FBG levels, remains one of the most widely used methods [21,22], the Metabolic Score for Insulin Resistance (METS-IR), which integrates multiple parameters, has been proposed as a marker that evaluates metabolic dysfunction within a broader framework [23].

Although the diagnostic and prognostic value of these indices has been extensively studied in adults, their clinical validity in adolescents has not yet been clearly established [24]. Adolescent PCOS may represent an early stage of metabolic dysfunction in adulthood; however, both metabolic alterations and systemic inflammatory responses in this age group may not yet have reached the levels typically seen in adults [25,26]. Moreover, puberty-related physiological insulin resistance and hormonal variability may interfere with the discriminatory performance of these markers. Consequently, the direct application of threshold values established in adult populations may increase the risk of diagnostic misclassification in adolescents [27,28].

In this context, there is a clear need to define the clinical utility of composite biomarkers reflecting systemic inflammation and metabolic dysfunction in adolescents with PCOS. Establishing the diagnostic and predictive performance of these indices—many of which have shown promising results in adult populations—in adolescents is of considerable importance for the early stratification of cardiometabolic risk. Accordingly, in the present study, we tested the hypothesis that parameters reported to be effective in adults may not demonstrate comparable performance in adolescents because of physiological dynamics specific to this developmental stage. The primary aim of the study was to comparatively evaluate the discriminatory performance of these composite indices in adolescent patients with PCOS. A further objective was to identify biomarkers that are easily accessible and feasible for routine clinical use, which could contribute to risk identification at an early stage.

## 2. Materials and Methods

### 2.1. Study Design and Sample

This study was designed as a retrospective, observational case–control study. Study data were obtained through a retrospective review of the electronic medical records of patients who presented to the Departments of Obstetrics and Gynecology and Pediatrics at Bandırma Onyedi Eylül University Faculty of Medicine Hospital between January 2023 and December 2025.

Patients younger than 18 years of age were included in this study. While the revised 2003 Rotterdam criteria [29] were referenced, to ensure diagnostic rigor and align with the updated international guidelines for adolescent PCOS [30], inclusion in the PCOS group was strictly limited to patients presenting with both menstrual irregularity and clinical or biochemical hyperandrogenism. Considering that polycystic ovarian morphology in adolescents can overlap with normal pubertal development, pelvic ultrasonography was systematically performed primarily to exclude other pelvic pathologies rather than as a primary diagnostic criterion. Menstrual irregularity was defined taking into account the time since menarche; specifically, oligo-/anovulation was considered as cycles longer than 45 days for 1 to 3 years post-menarche, and cycles longer than 35 days for more than 3 years post-menarche. Furthermore, hyperandrogenism, which forms the basis of the diagnosis, was evaluated in detail based on both clinical (presence of hirsutism assessed by the modified Ferriman–Gallwey score) and biochemical (total or free testosterone levels above the reference ranges) findings. The control group was randomly selected from adolescent girls who attended gynecology and pediatrics outpatient clinics during the same period for routine school health screenings, sports physical evaluations, or general wellness check-ups. To ensure an appropriate comparison, it was confirmed that these patients had experienced regular menstrual cycles for at least 1 year, and they were rigorously screened to exclude any underlying endocrine, inflammatory, metabolic, or other menstrual disorders.

To minimize potential confounding factors affecting metabolic and inflammatory parameters, patients with chronic conditions such as diabetes mellitus, thyroid disorders, Cushing’s syndrome, or adrenal disorders were excluded. In addition, lactating patients, those with acute or chronic systemic infections, and those receiving medications that could influence lipid metabolism or insulin sensitivity were also excluded from the study. Acute infection and inflammatory states were clinically excluded by the absence of leukocytosis and normal C-reactive protein (CRP) levels. To avoid confounding effects on inflammatory parameters, patients who experienced an acute infection within 4 weeks prior to blood sampling, those who used inflammatory, immunosuppressive, or hormonal medications within the previous 3 months, and those who provided blood samples during the luteal phase of their menstrual cycle were excluded from the study.

**Figure 1:**
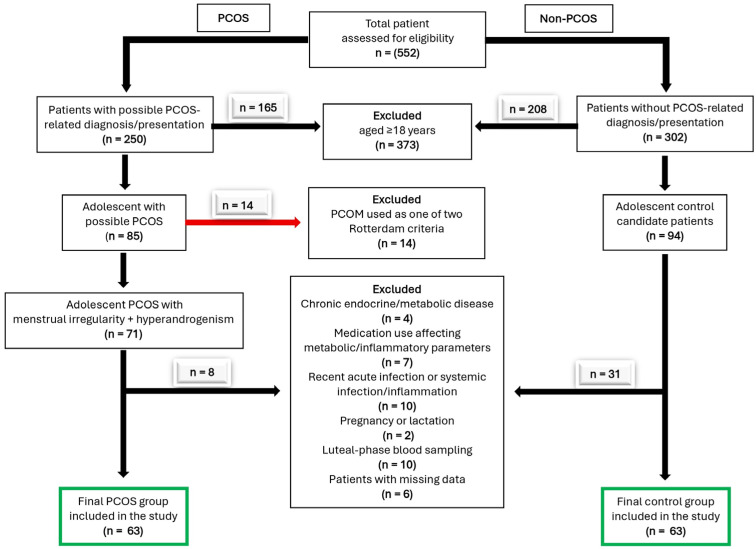
Flowchart of the study design. Note: PCOS, polycystic ovary syndrome; *n*, number of participants.

Convenience sampling was used for participant selection, and patients who met the eligibility criteria and had complete clinical and laboratory data were included in the study. During the initial retrospective screening of hospital records, a total of 85 adolescent girls with suspected PCOS were evaluated. To ensure full compliance with the new diagnostic guidelines, patients who did not concurrently meet both clinical criteria (menstrual irregularity and hyperandrogenism) and whose diagnosis relied on ultrasonographic findings, as well as those with missing data or who met other exclusion criteria, were excluded from this initial pool. After application of all inclusion and exclusion criteria, the final study population consisted of 63 adolescent females in both the PCOS and control groups (Figure 1).

### 2.2. Data Collection

The participants’ demographic characteristics, anthropometric measurements, and laboratory parameters were recorded using a structured data-collection form developed by the researchers. To ensure diagnostic consistency, all pelvic ultrasonography examinations were performed by a single designated experienced specialist in the gynecology outpatient clinic. Clinical evaluations, including the modified Ferriman–Gallwey scoring for hirsutism, were conducted by experienced specialists in both the gynecology and pediatrics outpatient clinics, strictly utilizing standardized visual reference charts to minimize observer-dependent variability. The variables collected included age, height, weight, FG, FI, TG, and High-Density Lipoprotein Cholesterol (HDL-C) levels. Additionally, complete blood count (CBC) parameters, specifically neutrophil, lymphocyte, monocyte, and platelet counts, were recorded using standard automated hematology analyzers to enable the calculation of inflammatory indices (SII, SIRI, and AISI). These laboratory measurements were obtained from routine biochemical analyses conducted following a fast of at least 8 h. Biochemical parameters were measured in serum obtained by centrifugation of blood samples collected in gel-sealed biochemical tubes, using an enzymatic colorimetric method on an automated biochemical analyzer (Cobas 6000, Roche Diagnostics, Mannheim, Germany). All analyses were performed in accordance with daily quality control procedures. All samples were analyzed in the same central laboratory of the hospital under strictly standardized conditions. The intra-assay and inter-assay coefficients of variation for all evaluated biochemical parameters were kept below 5%, ensuring high analytical reproducibility.

**Figure 2:**
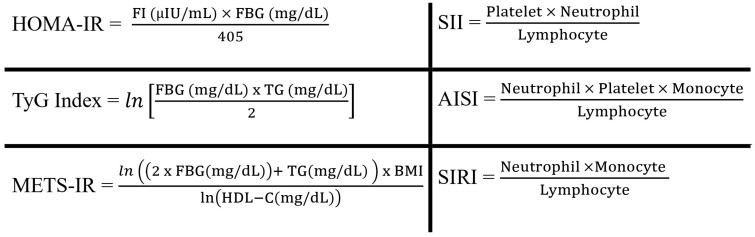
The calculation formulas for indices as defined in the literature. Note: HOMA-IR, homeostasis model assessment of insulin resistance; FI, fasting insulin; FBG, fasting blood glucose; SII, systemic immune-inflammation index; TyG, triglyceride-glucose index; TG, triglyceride; AISI, aggregate index of systemic inflammation; METS-IR, metabolic score for insulin resistance; BMI, body mass index; HDL-C, high-density lipoprotein cholesterol; SIRI, systemic inflammation response index.

Based on the recorded laboratory parameters, metabolic indices were calculated using standard formulas reported in the literature to assess the metabolic risk [31,32,33] (Figure 2).

All data were recorded anonymously, and personal identifiers were removed from the dataset prior to statistical analysis to ensure patient confidentiality.

### 2.3. Data Analysis

The data were analyzed using IBM SPSS Statistics for Windows, version 23.0 (IBM Corp., Armonk, NY, USA). Descriptive statistics including frequency, percentage, mean, standard deviation, median, and interquartile range were calculated. The suitability of the variables for a normal distribution was assessed using the Kolmogorov–Smirnov test. In comparing demographic and clinical characteristics according to the presence of PCOS, the Pearson chi-square test was used for categorical variables and the Mann–Whitney U test for continuous variables. In comparing the mean and median values of metabolic indices according to the presence of PCOS, Student’s *t*-test and the Mann–Whitney U test were used, depending on whether the distribution conformed to a normal distribution.

To evaluate the discriminatory power of the metabolic and inflammatory indices in distinguishing adolescent PCOS cases from healthy controls, Receiver Operating Characteristic (ROC) curves were plotted, and the Area Under the Curve (AUC), sensitivity, specificity, Positive Likelihood Ratio (LR+), and Negative Likelihood Ratio (LR−) were calculated to assess the ability of these indices to correctly distinguish individuals with PCOS. AUC values were classified as follows: 0.9–1.0 excellent, 0.8–0.89 good, 0.7–0.79 moderate, and 0.6–0.69 poor; values below 0.6 were considered inadequate [34]. In all statistical tests, a significance level of *p* < 0.05 was adopted.

## 3. Results

### 3.1. Demographic and Clinical Characteristics

No statistically significant differences were observed between the PCOS (*n* = 63) and control (*n* = 63) groups with respect to demographic and anthropometric characteristics (*p* > 0.05), indicating that the groups were comparable at baseline. However, when biochemical parameters were assessed, FBG levels were found to be significantly higher in the PCOS group compared to the control group (*p* = 0.001). In contrast, no significant differences were observed between the groups in terms of HDL-cholesterol, TG and FI levels. Additionally, no significant differences were found between the groups regarding basic hematological variables, including neutrophil, lymphocyte, monocyte, and platelet counts (*p* > 0.05) (Table 1).

### 3.2. Comparison of Metabolic and Inflammatory Indices

In the comparative analysis of composite indices, only the TyG index was found to be statistically significantly higher in the PCOS group compared to the control group (*p* = 0.028) among the metabolic markers assessed. No significant difference was observed between the groups in the HOMA-IR and METS-IR values, which are traditional and other composite indicators of insulin resistance. Similarly, no statistical difference was observed between the PCOS and control groups in terms of the SII, SIRI, and AISI indices, which reflect systemic inflammation (Table 2).

### 3.3. Logistic Regression Analysis

In the binary logistic regression analysis conducted to assess the association between the TyG index and the presence of PCOS, age, BMI and the TyG index were included in the model. The results indicated that the model’s overall explanatory power was low (Nagelkerke R^2^ = 0.055). Whilst no significant effect of age or BMI on PCOS was detected, the association between the TyG index and PCOS showed borderline significance (OR: 2.066; 95% CI: 0.986–4.329; *p* = 0.055) (Table 3).

### 3.4. Diagnostic Performance: ROC Analysis

**Figure 3:**
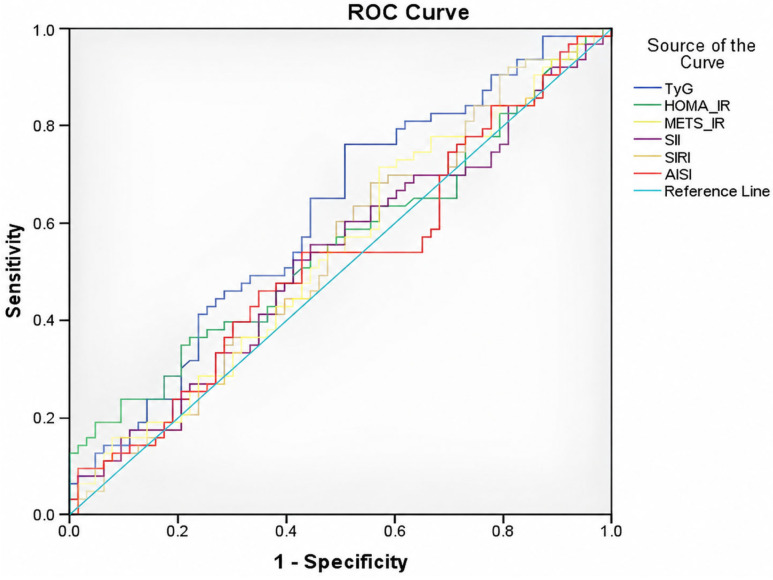
ROC curves of metabolic and inflammatory markers for PCOS discrimination. Note: ROC, receiver operating characteristic; AUC, area under the curve; PCOS, polycystic ovary syndrome; TyG, triglyceride-glucose index; HOMA-IR, homeostasis model assessment of insulin resistance; METS-IR, metabolic score for insulin resistance; SII, systemic immune-inflammation index; SIRI, systemic inflammation response index; AISI, aggregate index of systemic inflammation.

The results of the ROC curve analysis conducted to determine the performance of metabolic and inflammatory markers in distinguishing PCOS are presented in Table 4 and Figure 3. Among the parameters evaluated, only the TyG index was statistically significant in distinguishing PCOS from the control group (AUC: 0.614; 95% CI: 0.515–0.712; *p* = 0.028). However, this AUC value indicates that the index’s discriminatory power is weak. The AUC values of other metabolic and inflammatory markers remained around 0.50 and did not reach statistical significance. Overall, our findings suggest that the TyG index has limited discriminatory power in the age group studied, while other composite indices do not carry significant diagnostic value.

## 4. Discussion

In this study, the clinical and diagnostic value of composite indices (TyG, HOMA-IR, METS-IR, SII, SIRI, AISI) which reflects systemic inflammation and insulin resistance was comprehensively evaluated in terms of their ability to distinguish adolescent patients with PCOS. The findings are discussed below under relevant subheadings, alongside data from the current literature.

### 4.1. PCOS in Adolescence and Diagnostic Challenges

The clinical manifestations of PCOS vary over the reproductive lifespan. In adult women, PCOS has been extensively documented as a complex cardiometabolic disorder, frequently associated with established metabolic syndrome, severe insulin resistance, dyslipidemia, and chronic low-grade systemic inflammation [25,35]. However, the direct application of these adult-focused metabolic and inflammatory frameworks to adolescents overlooks the complex biological context of this period [36]. Adolescence is characterized by distinctive physiological features, transient physiological insulin resistance, and marked hormonal fluctuations. This can lead to the physiological emergence during adolescence of symptoms such as irregular menstrual cycles, acne and even biochemical changes that reflect the classic diagnostic criteria for PCOS [37]. Consequently, standardized diagnostic thresholds and reference ranges developed for adult cases may not provide sufficient sensitivity and specificity in the adolescent population, potentially resulting in diagnostic misclassification [38,39,40,41]. The findings of our study suggest that this limited clinical utility may be attributable to the metabolic and inflammatory profile of adolescence, which is not yet fully established and appears to be more limited and heterogeneous.

In the context of refining diagnostic criteria, the recent literature has critically examined the potential of serum anti-Müllerian hormone (AMH) as a surrogate marker for polycystic ovarian morphology. AMH demonstrates a strong correlation with ovarian morphology and remains stable across the menstrual cycle, suggesting it could improve diagnostic accuracy. Nevertheless, its routine diagnostic application, particularly in adolescents, is currently hindered by assay variability, the absence of standardized age-specific cut-offs, and the influence of confounding factors such as obesity and ethnicity. Therefore, incorporating such stable biomarkers into adolescent PCOS diagnostic criteria warrants further standardization [42,43,44].

### 4.2. The Power of Metabolic Indices and Glucose Metabolism

In our study, although no statistically significant difference in BMI was observed between the groups, the finding of higher FBG levels in the PCOS group suggests that metabolic dysfunction in adolescents may arise independently of overt obesity or apparent anthropometric alterations. This finding further indicates that indicators of early disturbances in glucose homeostasis may be detectable long before clinically significant anthropometric abnormalities become evident. Although the metabolic risk associated with PCOS is frequently attributed primarily to adiposity in the literature [45,46,47], our results highlight a more complex pathophysiological mechanism during adolescence. Indeed, the available evidence indicates that insulin resistance and impaired glucose tolerance are not limited to overweight or obese individuals but can be commonly observed in adolescents with PCOS across the entire BMI spectrum [36,48,49]. Accordingly, clinical risk assessment strategies based solely on BMI may be insufficient for early diagnosis in this age group. To ensure timely recognition of adolescent PCOS cases irrespective of weight status, biochemical parameters should be evaluated alongside anthropometric measurements [50,51].

In relation to glucose dynamics, it is noteworthy that, among the metabolic composite indices examined, the TyG index was the only one found to be significantly higher in the PCOS group. The TyG index, which is used to estimate insulin sensitivity, may be more sensitive in reflecting metabolic dysfunction during these early biological stages, when insulin resistance has not yet fully manifested itself [49,52]. Although statistically significant in univariate analysis, the weak diagnostic discrimination (AUC = 0.614) observed in the ROC analysis suggests that the TyG index might at best serve as a preliminary warning signal for metabolic changes rather than a robust marker [53,54]. Furthermore, it failed to remain an independent predictor of PCOS after adjusting for age and body mass index (BMI) in the logistic regression model. Consequently, given these limitations, the TyG index cannot be recommended as a primary diagnostic tool in this age group, but rather as a complementary indicator of early metabolic dysfunction [55,56].

Conversely, the fact that no significant difference was observed between groups in the HOMA-IR and METS-IR values [57,58]—which are well-validated markers of insulin resistance in adult women—highlights the unique challenges of quantifying metabolic burden in an adolescent population. In adults, the discriminatory power of these indices is enhanced by the cumulative effects of metabolic stress as the duration of the illness increases [23,59]. However, the metabolic profile during adolescence becomes more complex due to age-specific transient physiological insulin resistance, hormonal fluctuations and marked phenotypic variability, and the discriminatory power of these established biomarkers is reduced [60]. In this context, adolescent PCOS should be regarded not merely as an early manifestation of adult PCOS, but as a distinct clinical stage characterized by its own unique biological and metabolic dynamics [61]. Consequently, the clinical utility of markers such as HOMA-IR and METS-IR—which are standard in adults—should be reassessed in adolescents using an age-specific approach that considers the developmental context of adolescence [3].

### 4.3. The Role of Inflammatory Markers

In particular, the SII, SIRI, and AISI composite inflammatory indices failed to demonstrate significant discriminatory power in both group comparisons and ROC analyses. While this could initially imply that systemic inflammatory activity in adolescent PCOS may not yet be fully established, this interpretation must be approached with caution. This is because these systemic inflammatory indices are derived solely from routine complete blood counts and may not possess adequate sensitivity to capture the subtle, early-stage inflammatory processes occurring in adolescent PCOS. In adult PCOS populations, the clinical significance of these composite indices is invariably linked to the cumulative effects of metabolic stress, the persistent burden of obesity and enduring insulin resistance, all of which tend to become more entrenched and pronounced as the duration of the disease increases [62,63].

The relatively short duration of the disease in adolescence, combined with significant phenotypic heterogeneity and the confounding effects of age-specific physiological, immunological and hormonal variations, is likely to limit the discriminatory capacity of these markers [10,64]. Adolescence is a period characterized by dynamic developmental changes; consequently, in these patients, the inflammatory response is a highly variable process that is sensitive to both chronological age and the stage of disease progression, rather than a static or chronic condition [65]. Our findings do not rule out the role of inflammation in the pathophysiology of PCOS; rather, they support the view that systemic inflammation is a dynamic process dependent on age, disease stage and the accumulation of metabolic burden [66,67]. We therefore suggest that the diagnostic value of these markers should be interpreted within an age-specific perspective. Additionally, the lack of significant differences in SII, SIRI, and AISI may be partially attributed to the relatively small sample size of our study. This constraint may have limited the statistical power required to detect subtle but clinically meaningful differences in these inflammatory indices. Therefore, our findings may reflect insufficient statistical power rather than a true biological absence of low-grade inflammatory activity in this heterogeneous adolescent population.

### 4.4. Strengths and Limitations

The methodological strength of our study lies in the use of a matched control group, which reduces the likelihood that the observed analytical differences are attributable to physiological variations in age or body composition. In addition, the simultaneous evaluation of multiple metabolic and inflammatory indices within the same cohort provides a distinctive and comprehensive contribution to the literature by helping to clarify which pathophysiological axis is more dominant in the early stages of the disease. Furthermore, while combining multiple metabolic and inflammatory indices could theoretically enhance diagnostic discrimination, we opted not to perform composite multivariable predictive analyses. Given that most individual indices (except the TyG index) demonstrated limited baseline discriminatory capacity in our cohort, and considering the relatively small sample size, integrating them into a combined model could increase the risk of statistical overfitting. Therefore, we evaluated these indices individually to provide a more conservative assessment of their clinical utility.

However, certain limitations should be considered when interpreting our study. The single-center nature of the study and the relatively small sample size may have made it difficult to statistically detect minor effects, given the wide variation in the biomarkers examined, and may have limited the generalizability of the findings. Furthermore, although these composite indices are readily accessible in clinical practice, they are merely indirect reflections of the underlying complex biological processes. Additionally, due to the retrospective nature of the study, detailed information regarding participants’ lifestyle factors, such as diet and physical activity levels, was unavailable. As these factors can significantly influence metabolic and inflammatory parameters, their unmeasured confounding effects represent a limitation of the present study. Moreover, the retrospective nature of the participant selection process inherently carries a potential risk of selection bias, particularly concerning the control group. Although we applied rigorous inclusion and exclusion criteria based on detailed electronic medical records, the possibility of unmeasured confounders affecting internal validity cannot be entirely excluded. Also, hirsutism scoring was performed by multiple clinicians. Although standardized visual reference charts were used, the inherent subjectivity of the modified Ferriman–Gallwey system and the absence of a single examiner introduce a potential for observer variability, which represents a limitation of our clinical assessments. Consequently, this inter-observer variability may have compromised not only the precise classification of hyperandrogenic phenotypes but also the overall diagnostic accuracy, particularly for patients whose inclusion relied primarily on clinical manifestations. In addition, the retrospective design and relatively small sample size of our study present certain analytical constraints. Because comprehensive data on specific endocrine variables (such as androgen concentrations, sex hormone-binding globulin, LH/FSH ratio, and anti-Müllerian hormone) and continuous clinical severity measures were not uniformly available for all participants, we were unable to perform deeper evaluations of disease phenotypes or correlation analyses with clinical severity. Additionally, these methodological constraints restricted our ability to conduct robust stratified analyses based on obesity status, hyperandrogenism severity, or time since menarche. Consequently, evaluating subgroup-specific patterns and their correlations with metabolic and inflammatory markers remains a critical area to be comprehensively explored in future prospective studies with larger cohorts.

## 5. Conclusions

The finding of higher FBG levels and TyG indices in the PCOS group indicates that early metabolic changes can be detected during adolescence, whereas the evaluated systemic inflammatory indices did not show significant discriminatory power. However, although the TyG index was statistically higher in the PCOS group, its weak discriminatory capacity and lack of independent predictive value following adjustment substantially restrict its clinical utility. This limitation, combined with the failure of other composite metabolic and inflammatory indices to demonstrate clinically meaningful performance, does not support the use of these biomarkers as reliable standalone diagnostic tools in adolescent PCOS. Therefore, there is a need for prospective, multicenter studies with larger sample sizes and longitudinal designs to enable the more sensitive identification of early-stage risk and to validate the predictive utility of these biomarkers over time in adolescent PCOS. Nonetheless, integrating these composite indices with routine clinical features or other emerging diagnostic modalities may offer a more nuanced approach to cardiometabolic risk stratification in this population.

## Figures and Tables

**Table 1:** Comparison of demographic, anthropometric, biochemical, and hematological characteristics between the control and PCOS groups.

Variable	Control Group (*n* = 63) Mean ± SD or Median (25th–75th Percentile)	PCOS Group (*n* = 63) Mean ± SD or Median (25th–75th Percentile)	Test Statistic	p *
Age (years)	15.97 ± 0.98	15.94 ± 0.91	t = 0.188	0.851
BMI	25.73 ± 6.57	26.13 ± 6.66	t = −0.339	0.735
FBG (mg/dL)	88.76 ± 7.92	94.30 ± 10.66	t = −3.311	0.001
HDL-C (mg/dL)	52.60 ± 11.13	52.23 ± 12.30	t = 0.173	0.863
TG (mg/dL)	76.00 (60.00–108.00)	87.00 (65.00–133.00)	Z = −1.469	0.142
FI (µIU/mL)	11.10 (8.40–16.30)	12.00 (7.50–19.90)	Z = −0.566	0.571
Neutrophil (×10^3^/µL)	3.96 (3.37–5.22)	4.53 (3.48–5.50)	Z = −1.134	0.257
Lymphocyte (×10^3^/µL)	2.45 (2.12–3.13)	2.49 (2.10–2.84)	Z = −0.505	0.614
Monocyte (×10^3^/µL)	0.58 (0.49–0.67)	0.55 (0.47–0.66)	Z = −0.405	0.685
Platelet (×10^3^/µL)	320.00 (275.50–379.50)	309.00 (269.00–359.50)	Z = −0.881	0.378

Note: FBG, fasting blood glucose; TG, triglyceride; FI, fasting insulin; BMI, body mass index; HDL-C, high-density lipoprotein cholesterol; PCOS, polycystic ovary syndrome; SD, standard deviation; *t*, *t*-test statistic; Z, standardized Mann–Whitney U test statistic; *p*, *p*-value; *n*, number of participants. Data are presented as mean ± SD for normally distributed variables and as median (25th–75th percentile) for non-normally distributed variables. * *p* values were calculated using the independent samples *t*-test for normally distributed variables and the Mann–Whitney U test for non-normally distributed variables.

**Table 2:** Comparison of metabolic and inflammatory markers between the control and PCOS groups.

Variable	Control Group (*n* = 63) Mean ± SD or Median (25th–75th Percentile)	PCOS Group (*n* = 63) Mean ± SD or Median (25th–75th Percentile)	Test Statistic	*p* *
Metabolic markers				
TyG	8.16 (7.86–8.45)	8.26 (8.09–8.62)	Z = −2.201	**0.028**
HOMA-IR	2.35 (1.66–3.72)	2.69 (1.78–4.35)	Z = −1.078	0.281
METS-IR	36.21 ± 11.35	38.02 ± 11.23	t = −0.896	0.372
Inflammatory markers				
SII	517.74 (360.38–735.91)	569.17 (343.91–777.57)	Z = −0.559	0.576
SIRI	0.88 (0.61–1.29)	0.95 (0.69–1.37)	Z = −0.764	0.445
AISI	269.22 (176.58–480.86)	284.23 (182.13–496.54)	Z = −0.451	0.652

Note: AISI, aggregate index of systemic inflammation; HOMA-IR, homeostasis model assessment of insulin resistance; METS-IR, metabolic score for insulin resistance; PCOS, polycystic ovary syndrome; SII, systemic immune-inflammation index; SIRI, systemic inflammation response index; TyG, triglyceride-glucose index; SD, standard deviation; *t*, *t*-test statistic; Z, standardized Mann–Whitney U test statistic; *p*, *p*-value; *n*, number of participants. Data are presented as mean ± SD for normally distributed variables and as median (25th–75th percentile) for non-normally distributed variables. * *p* values were calculated using the independent samples *t*-test for normally distributed variables and the Mann–Whitney U test for non-normally distributed variables.

**Table 3:** Binary logistic regression analysis for the association between TyG and PCOS.

Variable	B	SE	Wald	*p*	OR (95% CI)
Age	−0.005	0.195	0.001	0.981	0.995 (0.679–1.459)
BMI	−0.012	0.029	0.178	0.673	0.988 (0.933–1.046)
TyG	0.725	0.378	3.693	0.055	2.066 (0.986–4.329)

Note: Model fit: Omnibus χ^2^ = 5.347, *p* = 0.148; Cox & Snell R^2^ = 0.042; Nagelkerke R^2^ = 0.055. BMI, body mass index; CI, confidence interval; OR, odds ratio; PCOS, polycystic ovary syndrome; SE, standard error; TyG, triglyceride-glucose index; Wald, Wald chi-square statistic; B, regression coefficient; *p*, *p*-value.

**Table 4:** ROC analysis of metabolic and inflammatory markers for discriminating PCOS.

Variable	AUC	SE	95% CI	*p* *
TyG	0.614	0.050	0.515–0.712	0.028
HOMA-IR	0.556	0.052	0.455–0.657	0.281
METS-IR	0.540	0.052	0.439–0.641	0.436
SII	0.529	0.052	0.427–0.630	0.576
SIRI	0.539	0.052	0.438–0.640	0.445
AISI	0.523	0.052	0.422–0.625	0.652

Note: AISI, aggregate index of systemic inflammation; AUC, area under the curve; HOMA-IR, homeostasis model assessment of insulin resistance; METS-IR, metabolic score for insulin resistance; PCOS, polycystic ovary syndrome; ROC, receiver operating characteristic; SE, standard error; SII, systemic immune-inflammation index; SIRI, systemic inflammation response index; TyG, triglyceride-glucose index. * *p* values test the null hypothesis that AUC = 0.50.

## Data Availability

The original contributions presented in this study are included in the article. Further inquiries can be directed to the corresponding author.
